# Monoclonal gammopathy exclusion in transthyretin amyloid cardiomyopathy: iStopMM estimated glomerular filtration rate-adjusted serum-free light chain ratio cut-offs

**DOI:** 10.1093/eurheartj/ehag250

**Published:** 2026-04-07

**Authors:** Muhammad Umaid Rauf, Yousuf Razvi, Joshua Bomsztyk, Ana Martinez–Naharro, Carol Whelan, William E Moody, Helen J Lachmann, Ashutosh D Wechalekar, Philip N Hawkins, Marianna Fontana, Julian D Gillmore

**Affiliations:** National Amyloidosis Centre, University College London, Royal Free Hospital, Rowland Hill Street, London NW3 2PF, UK; National Amyloidosis Centre, University College London, Royal Free Hospital, Rowland Hill Street, London NW3 2PF, UK; National Amyloidosis Centre, University College London, Royal Free Hospital, Rowland Hill Street, London NW3 2PF, UK; National Amyloidosis Centre, University College London, Royal Free Hospital, Rowland Hill Street, London NW3 2PF, UK; National Amyloidosis Centre, University College London, Royal Free Hospital, Rowland Hill Street, London NW3 2PF, UK; Institute for Cardiovascular Science, University of Birmingham, Edgbaston, Birmingham, UK; National Amyloidosis Centre, University College London, Royal Free Hospital, Rowland Hill Street, London NW3 2PF, UK; National Amyloidosis Centre, University College London, Royal Free Hospital, Rowland Hill Street, London NW3 2PF, UK; National Amyloidosis Centre, University College London, Royal Free Hospital, Rowland Hill Street, London NW3 2PF, UK; National Amyloidosis Centre, University College London, Royal Free Hospital, Rowland Hill Street, London NW3 2PF, UK; National Amyloidosis Centre, University College London, Royal Free Hospital, Rowland Hill Street, London NW3 2PF, UK

**Keywords:** ATTR-CM, Monoclonal gammopathy, eGFR, iStopMM, Non-biopsy diagnosis

The identification of monoclonal gammopathy (MG) in patients with suspected cardiac amyloidosis has critical diagnostic and prognostic implications. The validated and widely adopted non-biopsy diagnostic criteria (NBDC) for transthyretin amyloid cardiomyopathy (ATTR-CM), proposed by Gillmore *et al.* in 2016,^1^ require exclusion of a MG by serum and urine immunofixation and by serum-free light chain (sFLC) assay. However, the interpretation of serum free light chain (sFLC) ratio, which is a key component of this evaluation, is challenging in renal impairment. Different estimated glomerular filtration rate (eGFR)-adjusted reference ranges, measured by the Binding Site’s Freelite assay, for the normal polyclonal sFLC ratio in chronic kidney disease (CKD) have been proposed.^2,3^

Authors from the UK National Amyloidosis Centre (NAC) previously published eGFR-adjusted cut-offs for sFLC ratio, which have since been widely adopted in clinical practice and in clinical trials for ATTR-CM.^4^ More recently, findings from the iStopMM study, a large population based cohort in Iceland, proposed alternative eGFR-adjusted reference ranges for sFLC ratio, which also differ by not including urine immunofixation for exclusion of MG.^5,6^ However, the implications of using these new reference ranges within the NBDC for ATTR-CM remain undefined.

We compared the classification of MG at diagnosis using the NAC and iStopMM eGFR-adjusted sFLC ratio cut-offs in a cohort of 3315 patients referred to the NAC with suspected or proven cardiac amyloidosis (either by an endomyocardial biopsy or by NBDC) between 2016 and 2023. Complete biochemical data were available in all patients comprising sFLC assay (Freelite), serum and urine electrophoresis and immunofixation, and eGFR according to CKD epidemiology collaboration (2009 CKD-EPI creatinine) equation. Patients with a detectable monoclonal protein by serum immunofixation (*n* = 870), classified as having MG (MG+) under both definitions, were excluded from comparative analyses, leaving 2445 evaluable patients. Ethical approval for this study was obtained from the Royal Free Hospital Research Ethics Committee (IRAS 256590).

Applying iStopMM eGFR-adjusted sFLC ratio cut-offs without inclusion of urine immunofixation reclassified 16/2150 (0.7%) patients from MG− by NAC criteria to MG+ and 135/295 (45.8%) patients from MG+ by NAC criteria to MG− by iStopMM criteria. All 16 patients reclassified as MG+ by iStopMM criteria had low Kappa/Lambda (K/L) ratio (i.e. lambda bias) on sFLC assay, whereas the majority (124/135) of those who were classified as MG+ by NAC criteria but MG− by iStopMM criteria had a high K/L ratio (i.e. kappa bias) on sFLC assay reflecting the different eGFR-adjusted cut-offs between the two models.

It is pertinent that among 2134 patients classified as MG− by both NAC and iStopMM sFLC ratio criteria, six (0.28%) were shown to have biopsy-proven AL cardiac amyloidosis (AL-CA). This underscores an inherent limitation of the NBDC for ATTR-CM and the need to pursue a target-organ biopsy among patients in whom the clinical suspicion of AL-CA remains high despite absence of a biochemically detectable MG. Notably, among 16 patients who were MG+ by iStopMM criteria but were MG− by NAC criteria, no patient had AL-CA, and among 135 patients who were MG− by iStopMM criteria but MG+ by NAC criteria, six had AL-CA.

Among 135 patients who were classified as MG− by iStopMM criteria but MG+ by NAC criteria, the additional inclusion of urine immunofixation to the iStopMM eGFR-adjusted sFLC ratio cut-offs identified five patients with Bence–Jones proteinuria (BJP), including five of six patients with AL-CA. Of those five patients, one subject with AL-CA and a Perugini Grade 2 technetium-99m–labelled 3,3-diphosphono-1,2-propanodicarboxylic acid (DPD) scan would have fulfilled the non-biopsy diagnostic criteria for ATTR-CM had urine immunofixation not been included, along with four of the five remaining patients with AL-CA who would have been labelled as MG−. One patient with AL-CA had only a mild kappa bias (and no BJP), which was within the normal polyclonal range according to the iStopMM model but indicative of MG according to NAC criteria.

Overall, iStopMM and NAC eGFR-adjusted sFLC ratio cut-offs showed comparable negative predictive performance of 99.7% for AL-CA as long as urine immunofixation was included to exclude MG. However, their diagnostic profiles differed: iStopMM cut-offs exhibited higher specificity (97.3% vs 93.2%), thereby minimizing false-positive classifications of MG, but modestly lower sensitivity (84.1% vs 84.8%), slightly increasing the risk of false-negative MG classification, with comparable negative predictive value for AL-CA (*Figure 1*).

**Figure 1:**
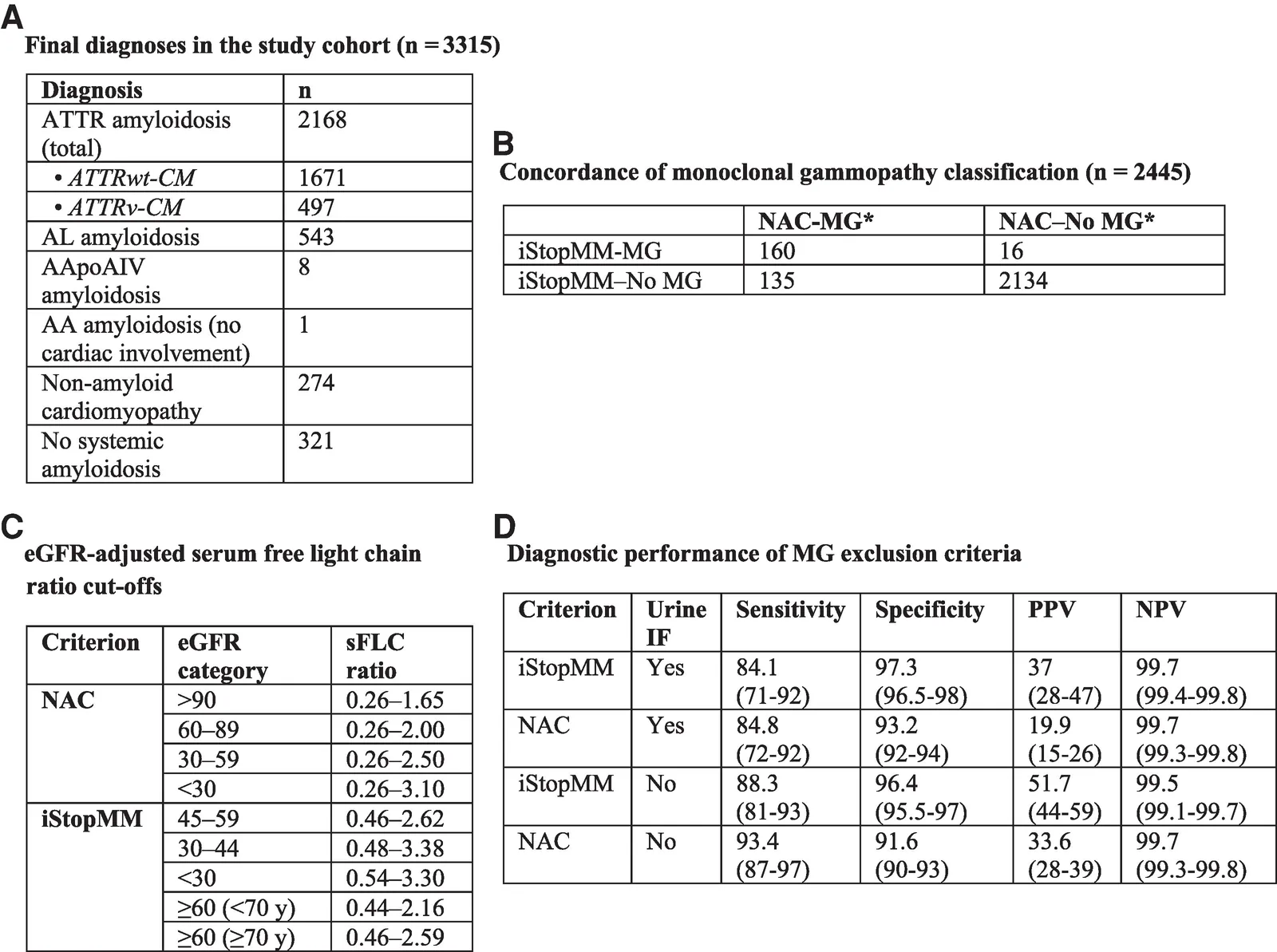
Estimated glomerular filtration rate-adjusted serum-free light chain ratio criteria for exclusion of monoclonal gammopathy. (*A*) Final diagnoses in the overall cohort of 3315 patients with suspected or proven cardiac amyloidosis. (*B*) Concordance of monoclonal gammopathy classification using the National Amyloidosis Centre and iStopMM criteria. (*C*) Estimated glomerular filtration rate-adjusted serum-free light chain ratio reference ranges. (*D*) Diagnostic performance of both criteria for exclusion of cardiac light-chain amyloidosis, stratified by inclusion or omission of urine immunofixation. Values are expressed as percentages with 95% confidence intervals (Wilson method). *National Amyloidosis Centre criteria include urine immunofixation for detection of the Bence–Jones protein. ATTR, transthyretin amyloidosis; ATTRwt, wild-type ATTR; ATTRv, variant ATTR; AL, light-chain amyloidosis; AApoAIV, apolipoprotein A-IV amyloidosis; MG, monoclonal gammopathy; sFLC, serum-free light chain; eGFR, estimated glomerular filtration rate; IF, immunofixation; PPV, positive predictive value; NPV, negative predictive value.

The selection of reference range for normal polyclonal eGFR-adjusted sFLC ratio has substantial impact on the diagnostic pathway in patients with suspected cardiac amyloidosis since the prevalence of MG increases with advancing age.^7^ Falsely attributing a patient as MG− when they do in fact have a subtle MG risks misclassifying them as having ATTR-CM on the basis of the NBDC when they may have AL-CA. Our data indicate that the NBDC for ATTR-CM are associated with a small inherent risk of misdiagnosis regardless of the chosen reference range but that as long as urine immunofixation is included among the diagnostic tests to exclude MG when using the iStopMM eGFR-adjusted sFLC ratio cut-offs, this risk is only slightly increased by switching from NAC to iStopMM eGFR-adjusted values. Moreover, the iStopMM eGFR-adjusted sFLC ratio cut-offs result in appreciably fewer patients with ATTR-CM requiring an endomyocardial biopsy due to being incorrectly classified as MG+ by NAC cut-offs, which aligns with the findings of the recently published study by Milani *et al*.^8^

Whilst the performance of the NBDC for ATTR-CM remains high with the use of iStopMM eGFR-adjusted sFLC ratio cut-offs, clinicians should remain cognisant that these thresholds were derived in a screening population and that, in order to preserve the specificity of the NBDC for ATTR-CM in a high pre-test probability setting, a full MG screen is essential. Our findings demonstrate that, as long as serum and urine immunofixation are included along with sFLC ratio within the MG screen, the high specificity of the NBDC for ATTR-CM is preserved and the risk of misclassification, including misdiagnosis of AL-CA, remains very low. Nonetheless, the authors continue to advise pursuing a target-organ biopsy in patients in whom clinical suspicion remains discordant with biochemical findings.

## Declarations

### Data availability

The authors agree to share publication-related anonymized patient data in accordance with the data sharing agreement. For original data, please email the submitting author (m.rauf@nhs.net) or the corresponding author (j.gillmore@ucl.ac.uk).
